# *Spirulina platensis* improves growth, oil content, and antioxidant activitiy of rosemary plant under cadmium and lead stress

**DOI:** 10.1038/s41598-023-35063-1

**Published:** 2023-05-17

**Authors:** Fatma Abd El Lateef Gharib, Eman Zakaria Ahmed

**Affiliations:** Department of Botany and Microbiology, Faculty of Science, Helwan University, Cairo, Egypt

**Keywords:** Biochemistry, Physiology

## Abstract

In the present study, a pot experiment was conducted to investigate the response of rosemary (*Rosmarinus officinalis* L.) plants to foliar application of *Spirulina platensis* at 0.0, 0.1, 0.2, and 0.4%; soil irrigation with heavy metals (Cd nitrate, Pb acetate, and Cd + Pb, each at 100 ppm), and *Spirulina platensis* at 0.1% + heavy metals. *Spirulina platensis* significantly improved growth parameters, oil yield/fed, photosynthetic pigments, and activity of superoxide dismutase (SOD), glutathione reductase (GR), catalase (CAT), and polyphenol oxidase (PPO) with a maximum promoting effect at 0.2% algal extract. On the other hand, heavy metal stress reduced growth criteria, photosynthetic pigments, and oil yield, while, significantly increased levels of antioxidant enzymes (SOD, CAT, GR) and corresponding non-enzymatic antioxidants (ascorbic acid, total antioxidant capacity, phenolics and flavonoids). Bioaccumulation factor (BF) and translocation factor (TF) indicated that Cd and Pb accumulated largely in the roots, with little transfer to the shoots. Nevertheless, compared with heavy metal treatments,* S. platensis* at 0.1% significantly increasing growth parameters, oil content, photosynthetic pigments, and the activity of non-enzymatic and enzymatic antioxidants, while, slightly reduced TF of Cd and Pb, alleviated membrane lipid peroxidation, and significantly lowered the content of malondialdehyde, hydrogen peroxide, and indole acetic acid oxidase (IAAO) activity in heavy metal (Cd, Pb, and Cd + Pb)-treated rosemary plants.

## Introduction

*Spirulina platensis* (Cyanobacterial family) is a biofertilizer and could potentially be utilised as a bioremediation agent against cadmium, chromium, and lead^1^. Arthrospira (*S. platensis*) has commercial importance for plants as a source of nutrients, proteins, vitamins, essential amino acids, fatty acids, polypeptides, phytohormones, and antioxidant compounds^2^. Foliar spraying of* S. platensis* increased tomato plant length, diameter, fresh biomass of shoot, and fruit^3^. Also, adding spirulina and Klamath algae to *Portulaca grandiflora* culture medium promotes growth, flowering, chlorophyll content, and NPK absorption^4^.

Heavy metals (HMs) pollution has now expanded across the globe, causing environmental and agricultural disruptions as well as posing major health risks to humans via the food chain. Cadmium (Cd) and Lead (Pb) are main pollutants, non-essential to metabolic functions. Cd is one of the most hazardous heavy metals due to its high mobility in the environment. In soils, Cd levels are increased by the application of sewage sludge, municipal garbage, and Cd-containing fertilisers. It can be easily absorbed by plants since it has an electronic configuration and a zinc-like valence state. Cd can affect cell biochemical mechanisms and structural characteristics by altering the behaviour of key enzymes in crucial pathways^5^. Cd can cause serious problems with photosynthesis, water interactions, and mineral intake by preventing necessary elements from being absorbed and translocated^6^. As a result, a complicated metabolic cascade within the cell, such as induction of antioxidant systems, can be triggered simultaneously with Cd-responsive gene transcription regulation^7^. On the other hand, Pb is a dangerous heavy metal and a well-known environmental pollutant. Pb can contaminate soils and plants through car exhaust, dust, fumes, and different industrial sources^8^. Pb binds tightly to the sulfhydryl groups of proteins and causes enzyme and structural protein deformation^9^. Lead ions can stimulate the formation of reactive oxygen species (ROS). An imbalance between the generation of harmful reactive intermediates and the ability of the cell to detoxify them eventually decreases oxidation–reduction enzyme activity^10^ and ultimately affects the yield and quality of plants.

Several studies have found that heavy metal (Cd and Pb) accumulation in plants causes numerous physiological and biochemical problems, and that using a biofertilizer can lessen the detrimental effects on some plants. Cd at low doses decreased chlorophyll content and growth of potato plant^11^ and rice plant^12^. Garg and Bhandari^13^ demonstrated that when bacteria or mycorrhizae interact with *Cajanus cajan* plants, the negative effects of Cd are decreased. Also, silicon modified ultra structure of chloroplasts, enhanced photosynthetic rate, and alleviates cadmium toxicity in maize^14^.

Rosemary (*Rosmarinus officinalis* L.) is a Mediterranean perennial herb with a long history. It's widely grown in Egypt and available all year. Rosemary (Lamiaceae family) is a frequently used spice in food processing as a natural source of antioxidants. Rosemary extract could be useful in pharmaceutical plant-based products. The essential oil derived from *R. officinalis* plants has a broad antimicrobial, antitumor, and antidepressant activities^15^. Oil yield, composition, and therapeutic value are all influenced by field farming practices, harvesting season, and plantation environment. However, heavy metal pollution can reduce productivity and the natural product values of rosemary plants. As a result, initiatives that optimise the utilisation of natural resources, reduce harmful element absorption, translocation and increase the production of safe plants are required. As mentioned above, *S. platensis* could improve plant growth and development and may alleviate heavy metal toxicity.

Therefore, the goal of this study was to improve rosemary plant tolerance against heavy metals (Cd and Pb) and focus on the effect of heavy metal stress on secondary metabolites production in this aromatic medicinal plant. Evaluating the effect on morphological traits and some metabolitc activities.

## Materials and methods

### Materials

Uniform cuttings of rosemary were generously donated by the Medicinal and Aromatic Plants Research Branch, El-Qanatir El-Khairiya, Horticulture Research Institute, Ministry of Agriculture, Cairo, Egypt. *Spirulina platensis* was kindly provided by the Algal Biotechnology Unit, National Research Centre, Dokki, Cairo, Egypt.

During the 2020/2021 season, a pot experiment was conducted at the Experimental Farm of Helwan University, Cairo, Egypt, Studies comply with local and national regulations Rosemary (*Rosmarinus officinalis*) plant was grown under foliar spray with *Spirulina platensis* and/or irrigation with heavy metals (i.e., Cd, Pb, Cd + Pb) throughout the two cuts of the experiment. Each pot was filled with clay loam soil, which contained 52.96% clay, 28.04% silt, 13.14% fine sand, and 5.86% coarse sand. In each pot, two uniform rosemary cuttings were transplanted. The earthenware pots were divided into three groups, each with 30–40 pots (30 cm in diameter and 28 cm in depth). The first group was separated into four subgroups and was twice foliarly sprayed with *S. platensis* solutions at concentrations of 0.0, 0.1, 0.2, and 0.4%, respectively, and irrigated with water. The second group was subdivided into three subgroups; each twice foliarly sprayed with distilled water and twice irrigated with one level of heavy metals (i.e., Cd as Cd nitrate; Pb as lead acetate; and Cd + Pb each at 100 ppm) for each group. The third group was subdivided into three subgroups; twice foliarly sprayed with *S. platensis* at 0.1% combined with twice irrigation with one level of heavy metals (i.e., Cd, Pb, Cd + Pb each at 100 ppm).* Spirulina platensis* treatments were employed prior to exposing rosemary plants to heavy metals stress. Three months after transplanting, the first spray was applied, and one week later, the second spray was done. Irrigation with heavy metals was carried out one week later, after the second spray.

Control plants were sprayed with distilled water just to cover the plant’s foliage completely till it dripped. Treatments were repeated two months after the first cut. The pots of the ten treatments were arranged in a completely randomised block design with six replicates, each replicate represented by two plants per treatment. Two cuttings (7 and 12 months from transplantation) were taken for experimentation.

Each pot was fertilized with one gram of potassium sulphate (48 percent K_2_O), ammonium nitrate (33.5 percent N), and calcium superphosphate (15.5 percent P_2_O_5_). These fertilizers were applied twice, 60 and 75 days after planting, and then again after the first cut. Irrigation was done at regular intervals based on weather conditions to keep the soil moisture content at field capacity.

### Growth criteria

At full blooming stage, the aerial portions (leaves and stems) were cut 5 cm above the soil surface, and plant growth parameters, i.e., plant height (cm), root length (cm), number of branches per plant, and fresh and dry weights of root, leaves, and stems (g plant^−1^) were recorded for the two cuttings. Plant samples were dried at 70 C in an oven with a drift fan until a constant dry weight was obtained. Fresh samples from different treatments were taken to determine essential oil content during the two cuts, as well as some metabolic and enzymatic activity during the first cut.

### Isolation of essential oil

During the two cuttings, essential oil (EO) from various treatments of rosemary was quantitatively determined by hydro-distilling aerial-fresh samples for 3 h with a Clevenger-type apparatus, and the oil yield per feddan was calculated on a dry weight equivalen.

### Determination of photosynthetic pigments

Photosynthetic pigments such as chlorophyll (a and b) as well as carotenoid concentration were assessed in fresh rosemary leaves and expressed as mg g^−1^ dry weight equiv. according to^16^.

### Analysis of Cd and Pb in soil and plants

Total levels of Cd and Pb in air dried soil and rosemary plants were determined in the Laboratory of Ecology, Faculty of Science, Helwan University, using Microwave Plasma Atomic Emission Spectroscopy (Agilent Technologies 4210 MP-AES). According to the manufacturer’s user manual, the instrument settings and operational instructions were adjusted.

The soil adhering to rosemary roots, the shoots and roots samples were taken individually from various pots and dried at 110 °C for 24 h. Samples were crushed and sieved through a 2 mm sieve after cooling. The shoots and roots of rosmary plants (0.5 g) were digested in a 15-mL acid mixture of HNO_3_: HCl (1:1, v/v) and heated on a hot plate until the digest turned clear. The digest was cooled, filtered, and diluted with twice-de-ionized water to a volume of 25 mL. Pb and Cd concentrations were measured and expressed in mg kg^−1^ on a dry matter basis.

Moreover, the bioaccumulation factor (BF), which assesses the ability of rosemary plants to accumulate Pb and Cd with respect to its concentration in the substrate^17^, was calculated in the following way:

BF = C_rr_/C_s_, where C_rr_ and C_s_ represent the concentrations of Cd and Pb in rosemary root and soil, respectively.

In addition, the translocation factor (TF), which determines the relative translocation of Pb and Cd metals from root to shoot of the plant species^17^, was estimated as:

TF = C_rs_/C_rr_, where C_rs_ and C_rr_ represent Cd and Pb concentrations in rosemary shoots and roots, respectively.

### Oxidative stress markers

#### Lipid peroxidation (malondialdhyde content)

As described by^18^, lipid peroxidation was measured in leaf tissue by the thiobarbituric acid (TBA) test, which determines malondialdhyde (MDA) as a product of lipid peroxidation. Leaf material (0.5 g) was homogenised in 10 ml of TBA reagent (18% Tri-chloroacetic acid (TCA) mixed with 0.45% TBA in a 1:2 ratio). After 15 min of incubation in a boiling water bath, the hot mixture was filtered through Whatman No. 42 filter paper, and the reaction was terminated by placing the reaction tubes in an ice bath. The samples were then centrifuged for 10 min at 6000 rpm, and the absorbance of the supernatant was measured at 532 nm. Non-specific absorbance at 600 nm was removed from the data at 532 nm. The concentration of MDA was measured in µmol g^−1^ d. wt. equivalent.

#### Membrane permeability

The permeability of cell membranes was measured using electrolyte leakage as described by^19^. Fresh leaves (2.5 g) were cut into 2-cm segments and placed in individual glass vials holding 25 mL de-ionized water after being washed with distilled water. After 30 min of soaking in water, the bathing solution's electrolytic conductivity (EC1) was measured with a conductivity metre (Model Ohm-419). The leaves were then brought to a boil, the bathing solution was cooled to room temperature, and the electrolytic conductivity was measured once more (EC2). The plasma membrane’s relative permeability was determined as follows:

$$ {\text{Relative }}\;{\text{permeability}}\left( \% \right)\; = \, \;\left( {{\text{EC1}}/{\text{EC2}}} \right) \, \times { 1}00 $$

#### Hydrogen peroxide content

The level of hydrogen peroxide (H_2_O_2_) was measured according to^20^. In an ice bath 0.5 g leaf tissue was homogenised in 5 mL of 0.1 percent (w/v) trichloroacetic acid (TCA). The homogenate was centrifuged for 30 min at 6000 rpm and 4 °C. 1.0 mL potassium iodide and 0.5 mL potassium phosphate buffer (10 mM, PH 7.0) were added to the supernatant (1.0 M). The absorbance of the supernatant was measured at 390 nm. Instead of plant extract buffer, 0.5 mL dist H_2_O was used for the blank solution. The concentration of H_2_O_2_ was calculated on a standard curve and the results were expressed as µmol g^−1^ d. wt. equivalent.

### Non-enzymatic antioxidants

#### Ascorbic acid

Estimation of ascorbic acid in rosemary was done using colorimetric assay kits acquired from Biodiagnostic Co., Cairo, Egypt, as described by^21^. One gram of fresh leaf tissue was extracted with 5 mL of TCA (10%). After centrifugation at 2000 rpm and 4 °C for 10 min, the supernatant obtained was used for estimation. The reaction mixture contained 200 μL of enzyme extract, 1.0 mL of buffer R1 (100 mM), and 2.0 mL 2,6-dichlorophenolindopheno R2 (DCPIP) at 1.0 mM. The assay mixture of the blank contained 200 μL of dist H_2_O instead of the enzyme extract. This mixture was well mixed and added to a clean quartz cuvette, and the absorbance of samples and blank were read in a spectrophotometer against dist water at 520 nm. The content of ascorbate in the samples was measured and expressed as (mg L^**−**1^).

#### Total antioxidant capacity

The total antioxidant capacity (TAC) of fresh rosmary leaf extract was measured according to^22^ using colorimetric assay kits purchased from Biodiagnostic Co., Cairo, Egypt. In brief, 0.02 mL of extract solution (1 mg mL^−1^) was well mixed with 0.05 mL R1 (substrate, H_2_O_2_) and incubated at 37 °C for 10 min, then added 0.5 mL working reagent (containing equal volumes of R2 (Chromogen) and R3 (Enzyme-Buffer)). The reaction mixture was mixed and incubated at 37 °C for 5 min. The assay of the blank contained 0.02 mL of dist. H_2_O instead of the enzyme extract solution. Then the absorbance of the blank and sample solution was read at 505 nm against dist. water. The antioxidant capacity of the extract was calculated and expressed as mM L^**−**1^ extract.

#### Total phenolic content

Air-dried powdered rosemary leaves (0.5 g) were extracted by stirring 25 mL of ethanol 70% at room temperature until the extraction solvent became colorless. As described by^23^, total phenolic content (TPC) was evaluated using Folin–Ciocalteu reagent and gallic acid as a standard. 0.5 mL filtered extracts were combined with 2.5 mL of Folin-Ciocalteu’s reagent (diluted with ethanol 1:10) and 2 mL of Na_2_CO_3_ (7.5%) and thoroughly mixed. After 15 min of incubation at room temperature, the absorbance of the resulting blue-colored solution was measured against a blank reagent at 765 nm using a Jenway 6405 UV–Vis spectrophotometer. The total phenolic content of the extract was measured in mg of gallic acid equivalents (GAE) per g of extract (mg GAE g^**−**1^ d. wt.).

#### Total flavonoids

The total flavonoid concentration (TFC) of the rosemary extract was determined using an aluminium chloride colorimetric assay^24^. 0.5 mL of sample solutions (0.5 g in 25 mL of ethanol 70%) were combined with 2 mL of dis. H_2_O, followed by 0.15 mL of 5% NaNO_2_ solution. After a 6-min incubation period, 0.15 mL of 10% AlCl_3_ solution was added and left to stand for 6 min before adding 2 mL of 4% NaOH. The mixture was diluted to 5 mL with methanol and thoroughly mixed. The absorbance was measured spectrophotometerically against a blank at 510 nm after a 15-min incubation period. The total flavonoid concentration was measured in mg of quercetin equivalents (QE) per gram of dry weight (mg QE g^−1^ d. wt.). Total flavonoids were calculated using a quercetin standard curve**.**

### Antioxidants enzymes

#### Superoxide dismutase (EC 1.15.1.1)

The superoxide dismutase (SOD) enzyme was assayed as U g^−1^ of fresh leaf tissue as described by^25^, using a superoxide dismutase Biodiagnostic ready kit purchased from Biodiagnostic Co., Cairo, Egypt. For extraction, 0.25 g of fresh leaf tissue was homogenised in 5 mL of cold 100 mM potassium buffer (pH 7.0), then centrifuged for 15 min at 4000 rpm and 4 °C. In a glass tube, add 0.5 mL of cold absolute ethanol/chloroform (60/40, v/v) to 1.0 mL of supernatant. Mix thoroughly for at least 30 s, then centrifuge for 10 min at 4000 rpm and 4 °C. The supernatant obtained was used for an enzyme assay. Before use, 2 mL of 50 mM potassium phosphate buffer (pH 8.5), 1 mM nitrobluetetazolium, and 1 mM NADH were mixed in a 10:1:1 ratio. Then, 0.2 mL of enzyme extract and 0.2 mL of distilled water were added to the assaying mixture. The assay mixture of the control contained the previous mixture without enzyme extract. This mixture was combined and placed in a clean quartz cuvette, and the reaction was started by adding 0.2 mL of 0.1 mM phenazine methosulphate (PMS) (diluted 1000 times before use) into the cuvette and mixing it immediately. The cuvette was inserted in the spectrophotometer for 5 min at 25 °C to record the increase in absorbance at 560 nm for control (∆ control) and the leaf tissue sample (∆ sample). SOD activity is expressed as (U g^−1^).

#### Glutathione reductase (EC.1.6.4.2)

A glutathione reductase (GR) enzyme was measured in leaf tissue as described by^26^ using a glutathione reductase Biodiagnostic ready kit for research reagents purchased from Biodiagnostic Co., Cairo, Egypt. 0.25 g of fresh leaf tissue was homogenised in 5 mL of cold potassium buffer (pH 7.5) containing 1 mM EDTA and centrifuged for 20 min at 4000 rpm and 4 °C. For the enzyme assay, the supernatant was employed, 2 ml of 100 mM potassium phosphate buffer (pH 7.5) containing 2 mM EDTA, 0.2 mL of oxidized glutathione (GSSG), and 0.2 ml of 2 mM nicotinamide adenine dinucleotide phosphate (NADPH) were added to the assaying mixture. The reaction is started by adding 0.1 mL of enzyme extract to the assaying liquid in a clean cuvette. The decrease in absorbance min^−1^ was recorded for 5 min at 340 nm. The activity of GR is expressed as unit L^−1^.

#### Catalase (EC 1.11.1.6)

According to^27^, fresh rosemary leaves were used for making enzyme extracts. In brief, 0.25 g of fresh leaf was homogenised in 5.0 mL of cold phosphate buffer (Na/K phosphate 0.1 M, pH 6.8), centrifuged for 10 min at 6000 rpm and 4 °C, and the supernatants were completed to a known volume and used for assaying the activity of catalase (CAT), polyphenol oxidase (PPO), and indole acetic acid (IAA) oxidase. Catalase was measured using a modified version of^28^ technique. To the reaction mixture, 1 ml of H_2_O_2_ (65 mM H_2_O_2_ in N/KP pH 7.4) and 0.2 ml of crude enzyme extrac were added to the reaction. The reaction was halted by adding 1 mL of ammonium molybedate (4 g L^−1^) after incubation for 4 min at 25 °C. The residual of H_2_O_2_ was followed by a decrease in absorbance at 405 nm. At the same time, a control was conducted in which the enzyme activity was stopped at zero time. The activity of catalase was expressed as µM H_2_O_2_ destroyed g^−1^ dry weight equivalent hour^-1^.

### Oxidative enzymes

#### Polyphenol oxidase (EC 1.10.3.1)

Polyphenol oxidase activity (PPO) was assayed according to^29^. The assay mixture contained 3 mL of buffered catechol solution (0.01 M catechol, freshly prepared in 0.1 M phosphate buffer, PH 6.0) and 1 mL of crude enzyme extract. Immediately, the colour intensity was read at 495 nm. Follow the change in absorbance for 30 s intervals up to 5 min. The enzyme activity was expressed as optical density g^−1^ d. wt. equiv. h^−1^.

#### Indole acetic acid oxidase (EC 1.2.3.7)

The activity of IAA oxidase was assessed using a modified approach of^30^. The reaction mixture (3 mL) contained 0.1 mL of 1 mM 2,6 dichlorophenol, 0.1 mL of 1 mM MnCl_2_, 0.4 mL of 0.1 mM potassium phosphate buffer pH 6, and 0.2 mL of 10 mM IAA. The reaction was initiated by adding 0.2 mL of enzyme extract, followed by 1 mL of p-dimethyl aminocinnamaldhyde (0.5% in 1 N HCl) as a control. The remaining reaction mixture was incubated in the dark. 1 ml of p-dimethyl aminocinnamaldhyde was added after 2 h and left in the dark for 1 h to develop the colour. The developed colour was spectrophotometrically monitored at 562 nm. The activity of an enzyme was expressed as optical density g^−1^ d. wt. equiv. h^−1^.

### Statistical analysis

For growth criteria, the data was expressed as the mean of six replicates, and for chemical analysis, the data was expressed as the mean of three replicates. Statistical analysis was carried out using one-way ANOVA followed by Duncan's Multiple Comparison Test in IBM Statistical Product and Service Solutions, SPSS Statistics for Windows, Version 21 at P 0.05, which was denoted as statistically significant for the means compared, using the least significant difference (LSD at 5 percent level).

## Results

### Growth parameters

**Figure 1:**
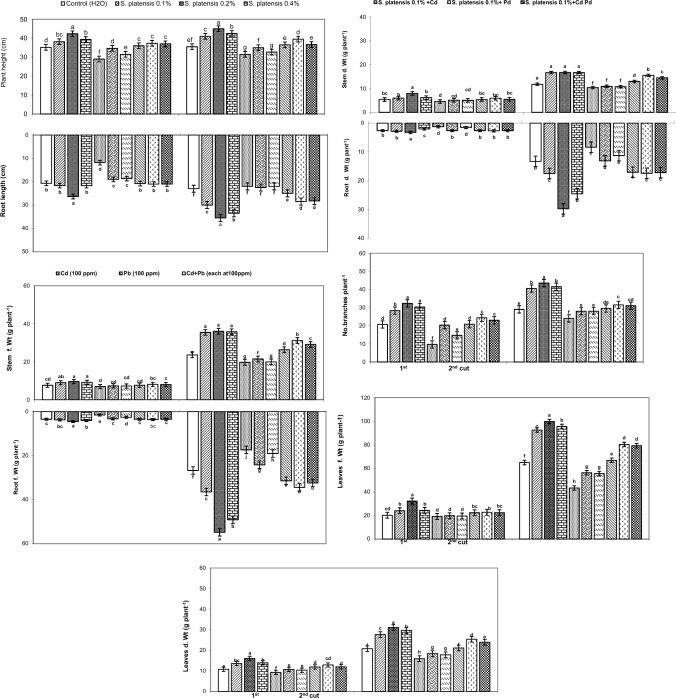
Effect of foliar spray with *Spirulina platensis* at 0.0, 0.1, 0.2 and 0.4%, irrigation with heavy metal (Cd as Cd nitrate; Pb as lead acetate; Cd + Pb each at 100 ppm), either alone or in combination* with S. platensis* at 0.1% on the vegetative growth of rosemary (*Rosmarinus officinalis*) plants during two cuttings. The results are expressed as means of six replicates. Different letters indicate significant differences between treatments (Duncan test, P ≤ 0.05) and Vertical bars represent LSD at 0.05 level.

Resultsshowed that foliar application of *Spirulina platensis* at 0.1, 0.2, and 0.4% on rosemary plants enhanced all growth criteria (i.e., plant height, root length, branch number per plant, fresh and dry leaf, stem, and root weights (g plant^−1^) compared to the untreated control plants. The increase in growth characteristics was significantly greatest with 0.2% of *S. platensis* during the two cuttings (Fig. 1).

On the other hand, Cd, Pb, and Cd + Pb stress significantly lowered the growth parameters of rosemary plants during the two cuttings compared to their relative controls and other treatments (Fig. 1). Cadmium stress significantly reduced the fresh (19.27, 43.45 g plant^−1^) and dry (9.32, 15.98 g plant^−1^) weights of leaves at the first and second cuttings, respectively, when compared to their related controls (20.26, 65.06 g plant^−1^) for f.wt and (10.81, 20.82 g plant^−1^) for d. wt.

Morover, *S. platensis* at 0.1% combined with heavy metals (Cd, Pb, Cd + Pb), reduced the harmful effect of heavy metals and improved growth parameters under stress conditions as compared to heavy metal treatments during the two cuttings. The most significant increases in growth metrics were seen in *S. platensis* at 0.1% + Pb, followed by* S. platensis* + Cd + Pb.

### Oil content

**Figure 2:**
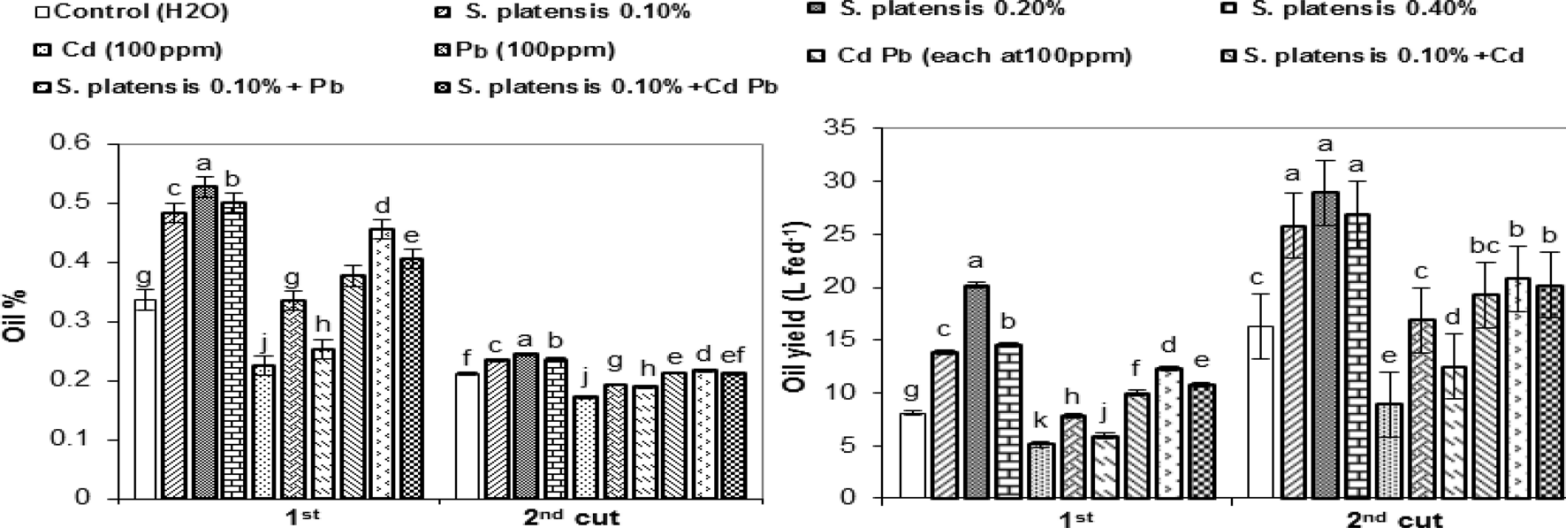
Essential oil percentage (mL 100 g^−1^) and oil yield (L fed^−1^) on a dry weight basis of rosemary (*R. officinalis*) plants as influenced by foliar spray with *Spirulina platensis* at 0.0, 0.1, 0.2, and 0.4%, irrigation with heavy metal (Cd as Cd nitrate; Pb as lead acetate; Cd + Pb each at 100 ppm), either alone or in combination with 0.1%* S. platensis*, at the 1st and the 2nd cuts. Statistical analysis was carried out using Duncan test. Vertical bars represent LSD at 0.05 level and different letters show significant variation at 0.05 P.

Figure 2 shows that the treatment of *S. platensis* alone enhanced essential oil (EO) percent and yield per feddan (L fed^−1^) on a dry weight equivalent basis of rosemary plants at both the 1st and 2nd cuts. When the two cuts were pooled together, the maximum increase in EO percent (40.58 and 34.27%) and oil yield (101.46 and 69.76% more than their corresponding controls) were obtained at 0.2 and 0.4% *S. platensis*, respectively.

However, Cd, followed by Cd + Pb, then Pb stress, recorded a decrease of 27.57, 19.40 and 3.61% in oil percentage, respectively, during the two cuts, as compared to the control. A more or less comparable trend was found with regard to the oil yield per feddan.

Considering the combined effect of *S. platensis* at 0.1% with heavy metals, application of *S. platensis* + Pb, followed by *S. platensis* + Cd + Pb, then *S. platensis* + Cd increased oil percent of the two cuts (increased 22.61, 12.57, and 7.74%) and oil yield (increased 35.61, 27.32, and 20.00% more than their controls), respectively. In most cases, as compared to untreated controls, the increase in oil content and yield were frequently significant (Fig. 2).

### Photosynthetic pigments

**Figure 3:**
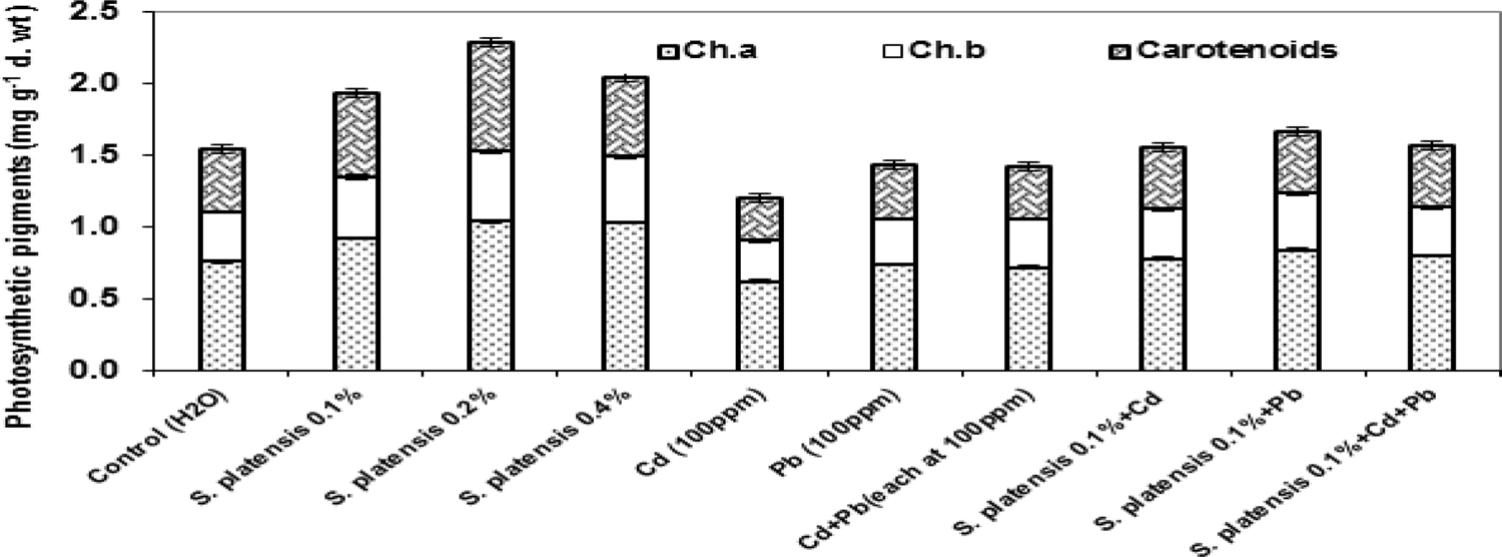
Effect of foliar spray with *Spirulina platensis* at 0.0, 0.1, 0.2, and 0.4%, irrigation with heavy metal (Cd as Cd nitrate; Pb as lead acetate; Cd + Pb each at 100 ppm), either alone or in combination* with S. platensis* at 0.1% on photosynthetic pigments (mg g^−1^ dry wt) in the leaves of rosemary (*Rosmarinus officinalis*) plants at first cut. Statistical analysis was carried out using Duncan test. Vertical bars represent LSD at 0.05 level and different letters show significant variation at 0.05 P.

Figure 3 demonstrates that when *S. platensis* was applied at any concentration or in combination with heavy metals, total photosynthetic pigments (TPP) in rosemary leaves were enhanced more than controls at the first cut. *S. platensis*, at 0.2 percent, was the most effective, followed by 0.4 percent, although heavy metals alone drastically reduced photosynthetic pigments.

Foliar application of *S. platensis* at 0.1, 0.2, and 0.4% significantly enhanced Chl a and b, carotenoids, and, as a result, TPP in the leaves of rosemary plants. The highest values of carotenoids and TPP (0.76 and 2.29 mg g^−1^ d. wt. equiv.) were achieved with 0.2% *S. platensis* compared to (0.43 and 1.54 mg g^−1^ d. wt. equiv.) for untreated control rosemary plants, respectively (Fig. 3).

On the other hand, Cd, Pb, and Cd + Pb stress significantly reduced Chl a and b, carotenoids, and TPP in rosemary leaves compared to untreated plants and other treatments (Fig. 3). Furthermore, the interaction of *S. platensis* at 0.1% with heavy metals increased these pigments in comparison to heavy metal stress, with the most effective treatment obtained with *S. platensis* + Pb.

### Uptake of Cd and Pb

The results of Cd and Pb analysis indicated variation in their concentrations in the root and shoot tissues of rosemary plants during the first cut (Table 1). The roots of rosemary accumulated Cd and Pb in higher concentrations than the shoots. The highest concentrations of Cd in the roots (3.65, 3.05, 2.70, and 2.40 mg kg^−1^) and shoots (1.40, 1.20, 1.00, and 0.90 mg kg^−1^) of rosemary were accumulated under Cd + Pb, Cd, *S. platensis* + Cd + Pb, and *S. platensis* + Cd, respectively compared with their respective Cd levels in control roots and shoots.

**Table 1:** Cadimum and lead concentrations (mg kg^−1^ dry weight) ± Std.

Treatments	Cd (mg kg^−1^ dry wt)			Pb (mg kg^−1^ dry wt)		
	Soil	Root	Shoot	Soil	Root	Shoot
Control (H_2_O)	0.042 ± 0.001	ND	ND	1.060 ± 0.006	0.65 ± 0.04	ND
*S. platensis* 0.1%	0.042 ± 0.001	ND	ND	1.058 ± 0.003	0.55 ± 0.02	ND
Cd (100 ppm)	2.518 ± 0.010	3.05 ± 0.03	1.20 ± 0.10	1.060 ± 0.003	0.60 ± 0.04	ND
Pb (100 ppm)	0.042 ± 0.002	ND	ND	5.176 ± 0.200	5.75 ± 0.33	0.77 ± 0.04
Cd + Pb (each at 100 ppm)	2.082 ± 0.022	3.65 ± 0.30	1.40 ± 0.20	5.032 ± 0.100	4.75 ± 0.12	0.75 ± 0.03
*S. platensis* 0.1% + Cd	1.518 ± 0.310	2.40 ± 0.20	0.90 ± 0.15	1.058 ± 0.033	0.58 ± 0.03	ND
*S. platensis* 0.1% + Pb	0.042 ± 0.003	ND	ND	4.496 ± 0.080	4.50 ± 0.25	0.35 ± 0.02
*S. platensis* 0.1% + Cd + Pb	2.052 ± 0.042	2.70 ± 0.50	1.00 ± 0.20	4.304 ± 0.101	3.00 ± 0.10	0.25 ± 0.02

Deviation in the soil, root and shoot of rosemary (*Rosmarinus officinalis*) plants foliar sprayed with *Spirulina platensis* at 0.0, 0.1, irrigated with heavy metal (Cd as Cd nitrate; Pb as lead acetate; Cd + Pb each at 100 ppm), either alone or in combination* with S. platensis* at 0.1% during the first cut.

ND = not detected.

On the other hand, the maximum accumulation of pb (5.75, 4.75, 4.50, and 3.00 mg kg^−1^) under Pb, Cd + Pb, *S. platensis* + Pb, and *S. platensis* + Cd + Pb, respectively compared with 0.65 mg kg^−1^ for their respective Pb levels in control roots, while the pb was slightly detected in the shoots under the same previous treatments (Table 1).

In comparison, the shoots and roots of rosemary accumulated higher Cd and Pb concentrations from heavy metals (Cd and Pb) irrigated soils than water irrigated soils. *S. platensis* foliar fertilizer reduced absorption of Cd and Pb in heavy metal (Cd, Pb, and Cd + Pb) irrigated soil (Table 1).

### Bioaccumulation and translocation factors

**Figure 4:**
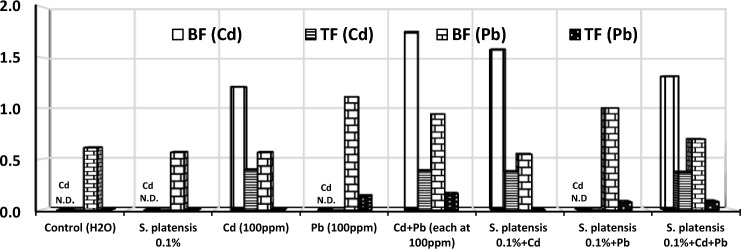
Cd and Pb bioaccumulation (BF) and translocation (TF) factors in rosemary (*Rosmarinus officinalis*) plants irrigated with heavy metals (Cd as Cd nitrate; Pb as lead acetate; Cd + Pb each at 100 ppm), either alone or combined with foliar spray with *Spirulina platensis* at 0.0 and 0.1% during the first cut.

The data of the bioaccumulation factor (BF) indicated that rosemary plants had a BF for Cd greater than one and higher than Pb in rosemary treated with heavy metals (Cd, and Cd + Pb) or *S. platensis* 1% + heavy metals-treated soils (Fig. 4). The highest BF (1.753) for Cd and Pb (1.111) were attained in Cd + Pb and Pd-treated soils, respectively.

As shown in Fig. 4, the translocation factor (TF) of Cd and Pb varied among all the treatments. The translocation factor of Cd was higher in rosemary when treated with Cd, Cd + Pb*,* singly or combined with* S. platensis* at 0.1% concentration. On the other hand, Pb and Cd + Pb, singly or combined with *S. platensis* 1% foliar application, increased the TF of Pb in rosemary, with the highest TF (0.393) for Cd and Pb (0.158) were recorded at Cd and Cd + Pb, irrigated soils, respectively.

Generally, the TFs were less than 1 for Cd and Pb treated with different treatments, suggesting a slight ability of rosemary to translocate Cd and Pb. Also, the TFs of Cd and Pb were reduced with the application of *S. platensis* 0.1% + heavy metals compared with heavy metals (pb, and Cd + Pb), singly.

### Oxidative stress markers

**Figure 5:**
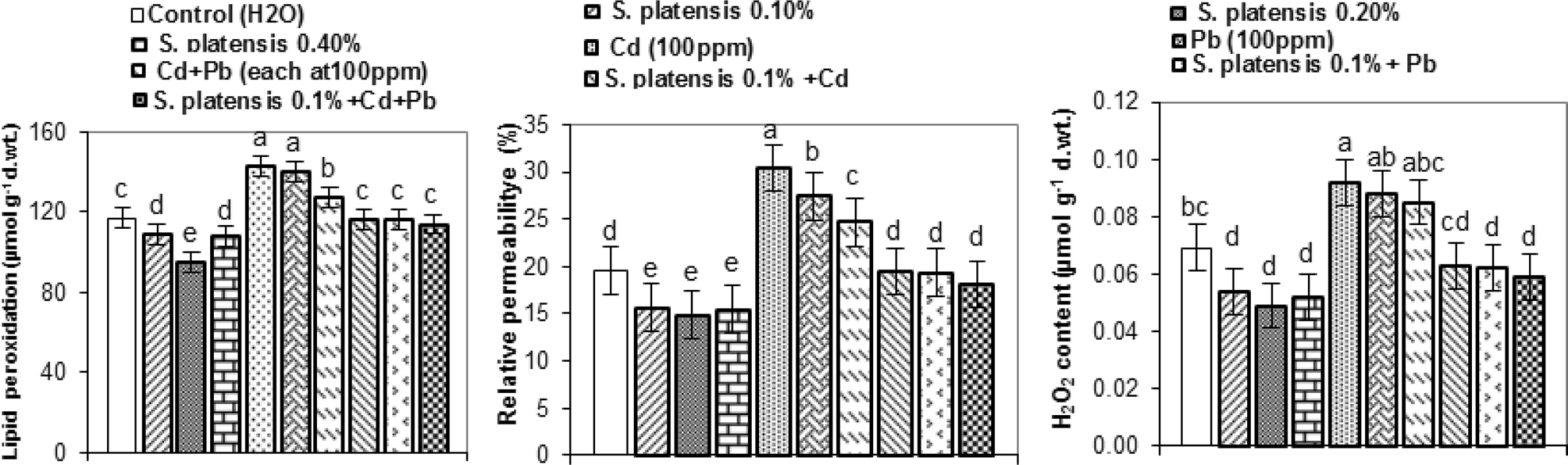
Lipid peroxidation (µmol g^−1^ d. wt. equiv.), relative permeability (%), and the content of hydrogen peroxide (µmol g^−1^ d. wt. equiv.) in leaf tissue of rosemary (*Rosmarinus officinalis*) plants, at the 1^st^ cut as influenced by foliar spray with *Spirulina platensis* at 0.0, 0.1, 0.2, and 0.4%, irrigation with heavy metal (Cd as Cd nitrate; Pb as lead acetate; Cd + Pb each at 100 ppm), either alone or in combination with 0.1%* S. platensis*. Statistical analysis was carried out using Duncan test. Vertical bars represent LSD at 0.05 level and different letters show significant variation at 0.05 P.

The data indicates that foliar application of* S. platensis*, singly or combined with heavy metal stress reduced levels of malondialdhyde (MDA) as a product of lipid peroxidation damage by free radicals, electrolyte leakage (EL) as a measure of cell membrane permeability, and hydrogen peroxide content, one of the most damaging forms of reactive oxygen species in the leaves of rosemary plants at first cut, relative to heavy metal treatments and controls (Fig. 5). The lowest values of MDA (95.0 µmol g^−1^ d. wt. equiv), relative permeability (14.86%), and H_2_O_2_ levels (0.049 µmol g^−1^ d. wt. equiv.) were recorded at 0.2% *S. platensis* compared with (117.0 µmol g^−1^ d. wt. equiv., 19.58%, and 0.069 µmol g^−1^ d. wt. equiv.) for their respective controls (Fig. 5).

On the other hand, heavy metals, especially Cd stress at 100 ppm, recorded the maximum values of peroxidative damage, relative permeability, and H_2_O_2_ content in rosemary leaves, being 143.0 µmol g^−1^ d. wt. equiv., 30.37%, and 0.092 µmol g^−1^ d. wt. equiv., respectively (Fig. 5).

### Non-enzymatic antioxidants

**Figure 6:**
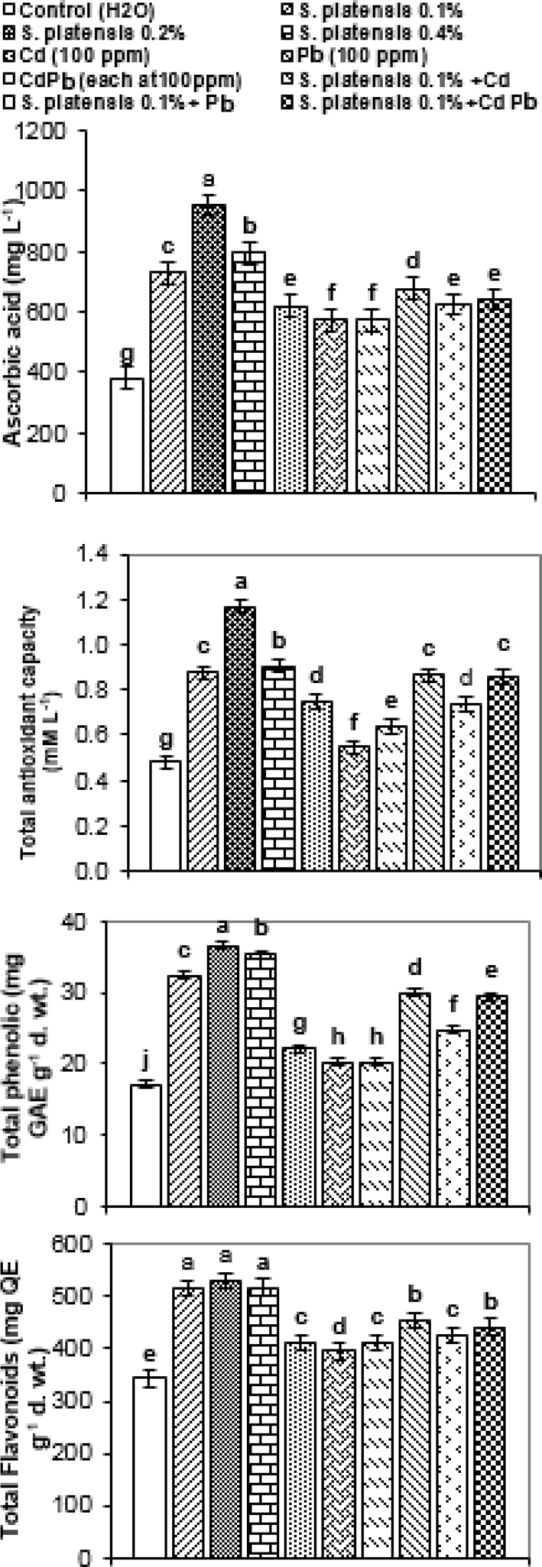
Changes in the content of ascorbic acid (mg L^−1^), total antioxidant capacity (mM L^−1^) of fresh leaf extract and total phenol compounds (mg gallic acid equivalents (GAE) g^−1^ d. wt.) and total flavonoids (mg quercetin equivalents (QE) g^−1^ d. wt.) of air dried rosemary (*R. officinalis*) plants at the first cut as affected by foliar spray with *S. platensis* at 0.0, 0.1, 0.2 and 0.4%, irrigation with heavy metals (Cd as Cd nitrate; Pb as lead acetate; Cd + Pb each at 100 ppm) either alone or in combination* with S. platensis* at 0.1%. Statistical analysis was carried out using Duncan test and different letters show significant variation at 0.05 P.

The results presented in Fig. 6 reveal that, in most situations, the trends in non-enzymatic antioxidants (total antioxidant capacity, ascorbic acid, total phenols, and flavonoids) under investigation were similar under foliar spray with *S. platensis* and/or heavy metal stress. Foliar application of *S. platensis* separately significantly increased the content of non-enzymatic antioxidants, relative to untreated control plants. The highest increase in ascorbic acid content (953.25, 794.37 mg L^−1^), antioxidant capacity (1.169, 0.906 mM L^−1^), phenolic (36.55, 35.32 mg GAE g^−1^) and flavonoid (530.65, 518.27 mg QE g^−1^) were obtained at 0.2, and 0.4% *Spirulina platensis*, respectively.

Moreover, heavy metal (Cd, Pb, and Cd + Pb) stress increased the above measured non-enzymatic antioxidants as compared with untreated control plants (Fig. 6).

Furthermore, the combination of *S. platensis* with heavy metals enhanced the content of total antioxidant capacity, ascorbic acid, total phenols, and flavonoids in the leaves of rosmary plants relative to their corresponding controls and heavy metals applied singly. The most promising increase in non-enzymatic antioxidants was obtained by foliar application of *S. platensis* at 0.1% + Cd, followed by *S. platensis* at 0.1% + Cd + Pb.

### Antioxidants and oxidative enzymes

**Figure 7:**
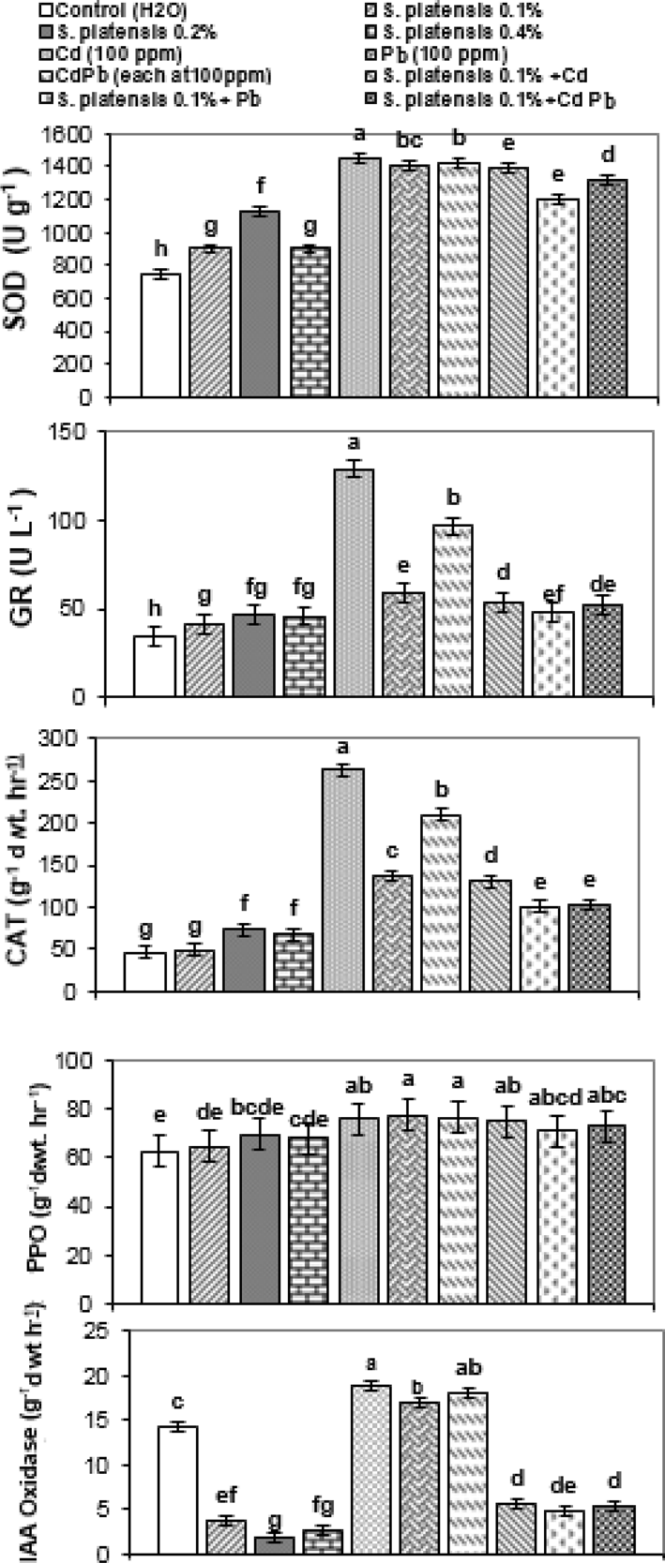
Changes in the activities of the antioxidant enzymes (superoxide dismutase (SOD) (U g^−1^), glutathione reductase (GR) (U L^−1^) and catalase (CAT) (g^−1^ d. wt. equiv. h^−1^) and two oxidative enzymes (polyphenol oxidase (PPO) and indoleacetic acid oxidase (IAAO) (g^−1^ d. wt. equiv. h^−1^) in fresh leaf tissue of rosemary (*R. officinalis*) plants at first cut as affected by foliar spray with *S. platensis* at 0.0, 0.1, 0.2 and 0.4%, irrigation with heavy metals (Cd as Cd nitrate; Pb as lead acetate; Cd + Pb each at 100 ppm), either alone or in combination* with S. platensis* at 0.1%. Statistical analysis was carried out using Duncan test. Vertical bars represent LSD at 0.05 level and different letters show significant variation at 0.05 P.

Figure 7 shows that foliar application of *S. platensis* at 0.1, 0.2, and 0.4% significantly increased antioxidant (superoxide dismutase (SOD), glutathione reductase (GR), and catalase (CAT)) activity as well as oxidative enzyme (polyphenol oxidase (PPO)) in rosemary leaves compared to controls at first cut. This stimulating effect was maximal for four measured enzymes at 0.2% *S. platensis* and thereafter declined; this is in contrast to the activity level of the oxidative enzyme indoleacetic acid oxidase (IAAO), which was reduced in response to the application of *S. platensis*, notably at 0.2 percent.

On the other hand, heavy metals significantly improved the activity of antioxidants (SD, GR, and CAT) and oxidative enzymes (PPO and IAAO) in rosemary leaves relative to untreated controls (Fig. 7).

Under heavy metal (Cd, Pb, and Cd + Pb) stress, an enhancing effect was observed in the activity of antioxidants (GR, SOD, and CAT) and oxidative enzymes (PPO) by foliar spray with *S. platensis* at 0.1%, compared with their corresponding controls, except for IAAO, where the activity decreased.

## Discussion

Application of *S. platensis* at concentrations of up to 0.4 percent, alone or under heavy metals Cd and Pb stress, considerably enhanced the vegetative growth, fresh and dry-matter content of rosemary plants, probably through plant growth-promoting substances present in algal extract such as auxins and cytokinins in addition to variable nutritive compounds and ions which enhanced growth criteria, leading to increased nutrient intake, activity of photosynthesis, and chlorophyll accumulation at both 1st and 2nd cutts. In this regard, the growth-promoting effects of *Arthrospira* spp. is not only linked to their hormone content, but also the nutrients in the extracts are easily absorbed by the leaves via stomata and cuticle hydrophilic pores. Foliar spraying of* S. platensis* increased plant length, diameter, fresh biomass of shoot and fruit in tomato plants at 3% aqueous extract, improved root dry matter, flower dry and fresh weights in petunias plants at 10 g L^−1^ *Arthrospira platensis* and *Scenedesmus* spp. hydrolysates^31^, and promoted the growth and flowering of *Portulaca grandiflora* plants^4^. Heavy metals (Cd, Pb, and Cd + Pb) stress showed a pronounced and substantial drop in rosemary plant vegetative growth,suppression inplant growth under heavy metal conditions may be a result of reduction in chlorophyll cintent by direct or indirect suppression in chlorophyll synthesis^32^, distortion of the chloroplast^33^. or the suppression of nutrient absorption^34^.This also agree with^35^ who reported that Low uptake of N, P, and K in bitter gourd under Cd toxicity might be a major factor for reduction in yield, fruit length, and fresh weight. In this respect, cadmium promoted a significant reduction in biomass production, height, root parameters, and inhibited the production and expansion of leaves in potato (*solanum tuberosum*) plants at 50 µM^11^, and inhibited growth of wheat plants by accumulation of Cd in plant tissue^36^. Pb significantly reduced shoot height, root length, and dry mass in *Leucaena leucocephala* seedlings at 300–700 µM Pb (NO_3_)_2_^37^. Intuitively, application of *S. platensis* under heavy metals stress revealed a much greater ability to promote the growth of rosemary plants compared with untreated plants. In this connection, the association of bacteria or mycorrhizae with plants showed a reduced deleterious effect of Cd, indicating other key biochemical and molecular mechanisms involved in Cd-stress tolerance^13^. The reduction of the negative effect of abiotic stresses observed in plants because of the interaction with cyanobacteria has been verified both directly, through its action in the soil, and indirectly, because of the activation of specific responses in plants^38^. Chen and Pan^39^ stated that living *Spirulina* cells had a high tolerance to lead and are good adsorbing agents. This may play arole in decreasing plant absorbance to Pb and Cd from soil. Indeed, besides its naturally fertilizing potential, Spirulina is able to release various bioactive molecules (polysaccharides, amino acids, phytohormones, etc.) that enhances plant growth and increase tolerance to biotic and abiotic stresses^40^.

The essential oil yield was generally higher during the second cut, compared to the first one, and the application of *S. platensis*, particularly at 0.2%, significantly increased the EO content and yield (L fed^−1^) of rosemary during the two applied cuttings compared to the corresponding control plants under normal conditions and in plants affected by heavy metals stress. This was accomplished by enhancing photosynthetic activity, fresh matter production plant^−1^, and EO biosynthesis. This was also proved by Gharib et al.^41^ on rosemary plants by foliar application of *Sargassum latifolium* extract at 0.2, and 0.4 percent which improved essential oil percentage and oil output per plant on a dry matter basis.The results obtained indicated that Cd and Cd + Pb stress, significantly reduced oil content on a dry weight basis for the two cuts due to reduction in photosynthesis, fresh aerial parts plant^−1^, essential oil biosynthesis, as the synthesis of essential oil in epidermal glands depends on providing photosynthetic carbon. In accordance with the present study, high toxic doses of Cd and Pb, disturb carbon translocations and consequently reduce essential oil percent of peppermint^42^ and significantly reduced oil yield of vetiver (*Vetiveria zizanoides*) grass in metal-contaminated soils^43^. In our investigation, Pb at 100 ppm had no effect on the essential oil output fed^−1^ in rosemary. Similarly, peppermint oil yield was not affected by the application of Pb (50–500 mg L^−1^) and Cd (2–10 mg^−1^)^42^. However,^44^ reported an enhancement in the essential oil percentage of certain aromatic plants under heavy metal stress.

Photosynthetic pigments in rosemary leaves were enhanced by the application of *S. platensis*, singly and under heavy metal stress (Fig. 3). *Spirulina platensis* might improve cell metabolic rate, delay senescence and Chl destruction and/or increase Chl biosynthesis by increasing N and Mg intake (structural component of Chl), resulting in increased Chl accumulation and a faster rate of photosynthesis. In this connection, Spirulina improved absorption of NPK and resulted in a significant increase in chlorophyll content in *Portulaca grandiflora*^4^*.* Also, the highest levels of chlorophylls and carotenoids were obtained in onion plants treated with *S. platensis* + cow dung, *Chlorella vulgaris* + cow dung, followed by *C. vulgaris, S. platensis,* cow dung, and then control plants, respectively^45^. In rosemary, treatment with heavy metals (Cd, Pb, and Cd + Pb) makedly decreased Chl a and b, carotenoids, and TPP in the leaves, which is a visual indicator of impaired growth more than control plants (Fig. 3). In this connection, Cd-negatively affected plant photosynthetic enzyme activity, pigment content, leaf stomatal conductance, induced chlorophyll degradation, and disorder in grana and thylakoids arrangements, resulting in decreased photosynthesis rate, and chlorophyll content in potato plants^11^. According to^37^ chlorophyll decreases in *Leucaena leucocephala* seedlings have been attributed to Pb (NO_3_)_2_ at 300–700 µM induced chlorophyll synthesis inhibition. In another research, stress of cadmium (10 and 40 mg kg^−1^) and lead (60 and 180 mg kg^−1^) significantly decreased chl a, chl b, chl a + b and carotenoid in *Matricaria chamomilla*^46^. Our results indicate that *S. platensis* at 0.1% might concomitantly alleviate the adverse effects of heavy metals, promoting plant biomass and enhancing the content of photosynthetic pigments in leaves. This is due to the fact that *S. platensis* can provide rosemary plants with some nutrients, vitamins, and hormones and also reduce Cd and Pb absorption from the soil, thereby improving plant growth and photosynthesis when compared to plants exposed to Cd and/or Pb stress. In this regard, providing soil amendments can provide plants with nutrients while minimising soil absorption of Cd and Pb, thereby promoting plant growth and improving photosynthesis^47^. Also, foliar application of *S. platensis* (100 mg L^−1^) extract significantly improved pigment content, photosynthetic activity, and transpiration rate under salinity stress in *Vicia faba* plants^48^.

In this study, Cd and Pb concentrations increased in both the shoots and roots of *R. officinalis* under Cd and/or Pb stress singly rather than when combined with *S. platensis*. The accumulations of Cd and Pb in the roots of rosemary plants were larger than those in the shoots. In this respect, more accumulation of Cd and Pb was observed in roots than in shoots of *Salix mucronata*^49^. However, the toxic effect of Cd was reduced by using a bacterium-containing biofertilizer in maize and sunflower plants^50^. The bioaccumulation factor (BF) generally indicates the transport of heavy metals from soil to the plant root, indicating the effectiveness of bio-available metal intake from the environment. According to our current investigation, the BF revealed the transfer and availability of Cd and Pb in rosemary, for soil treated with heavy metals (Cd, Pb, and Cd + Pb), singly and when combined with foliar spray with* S. platensis*. Rosemary plants can accumulate a higher Cd concentration in the roots than in the leaves, and the mobility of Cd in plants is higher than Pb, since it has an electronic configuration and a zinc-like valence state. The BF showed low transfer of Cd (1.316) and Pb (0.697) in rosemary treated with *S. platensis* + Cd + Pb compared with their respective values (1.753 and 0.944) for Cd + Pb treatment. Similar, low BF for Cd and Pb were obtained in wheat treated with four soil amendments (sepiolite, single superphosphate, triple superphosphate, and calcium magnesium phosphate) compared to the control. Also, the application of soil amendments is an effective phytostabilization for remediating Cd-and Pb-contaminated soil in ramie plants^51^. On the other hand, the translocation factor (TF) of Cd was greater than that of Pb in rosemary. due to its similar properties to Zn, but, Pb translocation was low because Pb was early recognised as a toxic molecule by plant roots and sequestered in the vacuole or in the cell walls of the root, resulting in low translocation in the shoots. In our research, the translocation factor (TF) was less than one for Cd and Pb in all treatments, indicating the potential of the rosemary plant for their phytostabilization in the root. Similar findings were reported by^45^ on Mentha species and^46^ on *Matricaria chamomilla* L.

Decrease in MDA production and relative permeability by application of *S. platensis* under normal and heavy metal stress suggests greater protection of rosemary plants from oxidative damage as compared to Cd and Pb treatments. Reduced lipid peroxidation (due to the inhibitory effect of *S. platensis*) favoured vegetative growth and the accumulation of essential oil (Figs. 1, 2) by protecting polyunsaturated fatty acids in the cell membrane from oxidative damage. Further, the amount of H_2_O_2_, one of the most harmful forms of reactive oxygen species, was reduced in rosemary plants treated with* S. platensis* and its combination with heavy metal treatments. This drop in H_2_O_2_ is caused by the activation of an H_2_O_2_ metabolising enzyme. Because of its high pKa, H_2_O_2_ can easily permeate the biological membranes and damage a variety of cellular organelles. A decrease in H_2_O_2_ generation suggests that the efficiency of redox processes has increased in the presence of *S. platensis*. In this regard, 50% hydrogen-rich water (HRW) + Cd(NO_3_)_2_ at1 μM significantly reduced the content of malondialdehyde, hydrogen peroxide, superoxide radical (O_2_^−^), relative electrical conductivity, lipoxygenase activity, plasma membrane permeability, and protect the membrane lipid stability in cucumber (*Cucumis sativus* L.) explants compared to Cd treatment^52^. Moreover, maximum production of H_2_O_2_, induced damage to MDA content and membrane integrity, was recorded under single Cd followed by Pb stress, which showed a 55.11 and 39.99% increase in electrolyte leakage, respectively, relative to control. Cd and/or Pb, treatments, cause a rise in malondialdehyde concentration, which indicates the production of free oxygen radicals and may result in changes in membrane fluidity, alterations in phospholipids, and an increase in electrolyte leakage (Fig. 5). In this connection, Cadmium stress induces overproduction of ROS and subsequent oxidative stress, with increase in the content of H_2_O_2_ and TBARS in cucumber explants^53^ and rice seedlings under Cd, lead (Pb) or Cd + Pb stress^54^. ROS can speed up lipid peroxidation, affecting cell membrane fluidity and permeability due to changes in membrane lipid composition^55^. On the other hand, any rise in H_2_O_2_ has severe effects on the damaged cell. In our investigation, Cd stress resulted in a 33.33% increase in H_2_O_2_ levels, while Pb stress resulted in a 27.54% increase over the control value (Fig. 5). A larger accumulation of H_2_O_2_ under stress indicates that H_2_O_2_ metabolising enzymes are not working properly. Cd poisoning cause electrolyte leakage, overproduction of hydrogen peroxide, and malondialdehyde content in wheat (*Triticum aestivum* L.) plants^56^.

Foliar spray with *S. platensis,* especially at 0.2%, significantly increased the content of non-enzymatic antioxidants (total antioxidant capacity, ascorbic acid, total phenols, and flavonoids), relative to untreated control rosemary plants. Similarly, a significant increase in total phenols and enhanced growth were obtained by *S. platensis* in onion plants^45^. Furthermore, heavy metal stress increased the measured non-enzymatic antioxidants as compared with untreated control rosemary plants (Fig. 6). During Cd stress, the rise in the production of non-enzymatic antioxidants, such as ascorbate and glutathione (GSH), as well as vitamins, flavonoids, and carotenoids, may have a valuable role in detoxify ROS produced under stress conditions^57^. According to^57^, total phenol level, ascorbic acid (AsA), α- tocopherol and carotenoids contents were all significantly higher in cadmium-exposed plant species. The rise in phenolics attributed to an increase in the activity of enzymes involved in phenolic metabolism, implying de novo phenolic synthesis in the presence of metal stress. However,^54^ found that Cd, Pb, and Cd + Pb treatments enhanced dehydroascorbate (DHA) level while decreasing AsA level and the AsA/DHA ratio in rice seedlings. Our results indicated that the most promising increase in non-enzymatic antioxidants (total antioxidant capacity, ascorbic acid, total phenols and flavonoids) was obtained by foliar application of *S. platensis* at 0.1% + Cd, followed by *S. platensis* + Cd + Pb in the leaves of rosmary plants, relative to their corresponding controls and single heavy metal stress. In this respect, treatments with *Spirulina* spp. extract increased the antioxidant capacity in *Vicia faba* under salinity stress^46^. Also, hydrogen-rich water (50%) alleviated oxidative stress caused by Cd (NO_3_)_2_ in cucumber (*Cucumis sativus* L.) explants and induced increases in a nonenzymatic protection system, such as AsA/DHA and GSH/GSSG ratio^53^.

*Spirulina platensis* foliar treatment, at any dose significantly raised the activity levels of superoxide dismutase (SOD), glutathione reductase (GR), catalase (CAT) and PPO in the leaves of rosemary plants, while, the opposite result was achieved with IAAO activity levels that dropped in response to *S. platensis* treatment, particularly at 0.2 percent compared with controls. In this connection, application of *Ascophyllum nodosum* extract elevated antioxidant levels in spinach leaves^58^. Under single Cd, Pb, and Cd + Pb stress, the activities of SOD, GR, CAT, and PPO were significantly enhanced in rosemary, which indicates the scavenging mechanism of the plants against the ROS produced in response to the metal stress. According to^59^, Cd induces several ROS, including the superoxide radical (O2•−), hydrogen peroxide, and hydroxyl radical (OH•), systems capable of preventing uncontrolled oxidation are induced or stimulated, including antioxidant enzymes such as SOD, GR, CAT, glutathione peroxidase (GPX), ascorbate peroxidase (APX), and other peroxidases, which detoxify H_2_O_2_^60^. Superoxide dismutase is the principal scavenger in active oxygen species detoxification in plants, converting superoxide to hydrogen peroxide and oxygen, protecting cells from superoxide-induced oxidative stress. In addition, CAT detoxifies H_2_O_2_ into H_2_O and O_2_. High cadmium stress (100 μmol L^−1^) increased GR 337.7%, SOD 688.6%, POD 72.2%, CAT 229.0%, and APX 226.5% activity in* Arachis hypogaea* L. leaves^59^, significantly increased activity of POD, PPO, and CAT in pea seedlings at 25–50 µM cadmium acetate concentrations^57^ and increased the activities of enzymatic antioxidant defense system (ADS) to some extent, while non-enzymatic ADS dramaticly decreased in *Capsicum annuum* under Cd and/or Pb stress^60^. However, the rise in PPO activity was also observed in pea seedlings under Cd-stress, and in both *Ceratophyllum demersum* L. and *Hydrilla verticillata* L. plants under Pb (5–80 µM) stress^56^. Our results indicated that under heavy metal (Cd, Pb, and Cd + Pb) stress, *S. platensis* treatment at 0.1% increased SOD, CAT, GR, and PPO activity, while significantly decreasing IAAO activity, as compared to control rosemary plants, and this may be related to differences in antioxidant enzyme expression to get rid of reactive oxygen species and increase plant tolerance in response to heavy metal stress. Avoiding oxidative stress and reestablishing redox homeostasis are critical in alleviating metal stress^54^. Under salinity stress, *S. platensis* decreased the large levels of SOD, POD, CAT, and GPX in *Vicia faba* as compared to salt-stressed plants^48^. Furthermore, under Cd stress, hydrogen-rich water treatment significantly reduced IAAO activity, ascorbic acid content, and reduced glutathione (GSH) in cucumber explants while significantly increasing the activity and related gene expression of ascorbate peroxidase, dehydroascorbate reductase, monodehydroascorbate reductase, and GR in comparison to Cd treatment. It also reduced oxidative damage by increasing the activities of PPO and POD^55^. Improvement in plant antioxidant capacity by application of *Spirulina* algae is agood indication of increase in rosemary plant tolerance toward heavy metal stress and overcome the damage may be caused by reactive oxygen species produced under stress.

## Conclusion

Environmental pollution by heavy metals increases by time due to development in industrial and agricultural aspects. *Spirulina platensis* (biostimulant) promoted rosemary (*R. officinalis* L) growth, physiological performance in terms of photosynthetic production, essential oil content, and was efficient in mitigating Pb and/or Cd toxicity. *Spirulina platensis* alleviated membrane lipid peroxidation, inhibited the production and accumulation of ROS through modulating the antioxidant defence system and promoted the reduction of Cd and Pb absorption and accumulation in rosemary. The TFs were less than 1 for Cd and Pb in rosemary irrigated with heavy metals (Cd, Pb, and Cd + Pb), and sligtly reduced with the application of *S. platensis* at 0.1%, which indicates low translocation. Therefore, this study indicates that foliar spray with *S. platensis* may be a viable novel strategy for agricultural improvement of medicinal and aromatic plants under normal and heavy metal (Cd, Pb, or Cd + Pb) stress.

## Supplementary Information

Supplementary Information.

## Data Availability

All data generated or analyzed during this study are included in this article. In addition to provided supplementary materials.
